# Supplementary material for: Morin Treatment Delays the Ripening and Senescence of Postharvest Mango Fruits
Source: Foods. 2023 Nov 24;12(23):4251. doi: 10.3390/foods12234251 (PMC10706440; doi:10.3390/foods12234251)
Supplement: Supplementary file 1 [file foods-12-04251-s001.zip › Supplementary material.pdf]

Supplementary material

Figure S1. Effect of 0.005% and 0.01% morin treatment on appearance characteristics of mango fruit during storage at 25±1℃after 18 days.

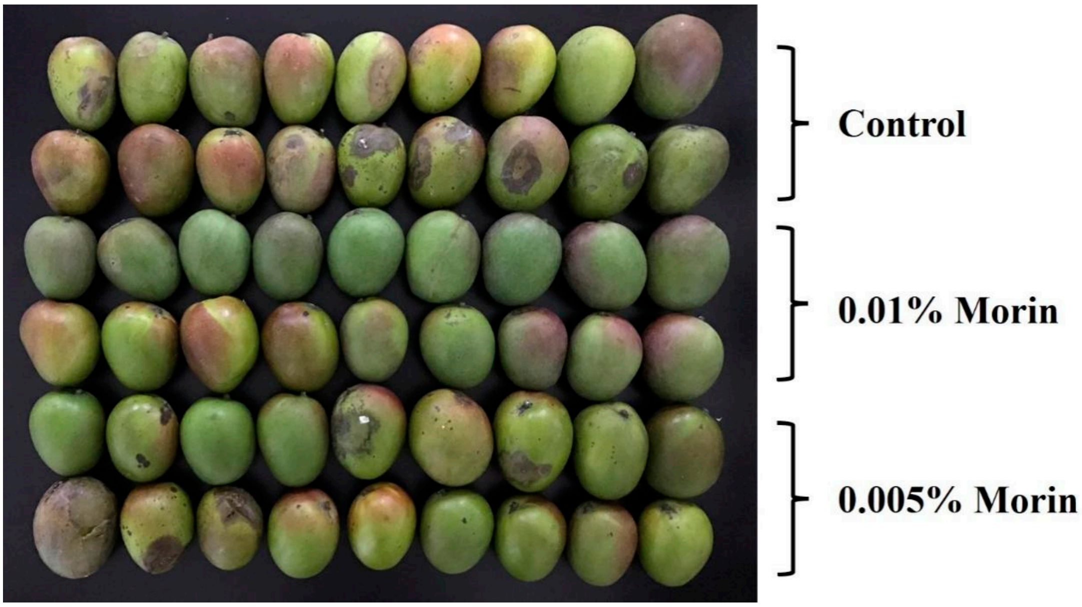

Table S1. Primers for Quantitative real-time PCR (qRT-PCR).

Gene name	Primer (5′-3′)	References
Actin-qPCR-F	GAGAGTTTTGATGTCCCTGCCATG	[26]
Actin-qPCR-R	CAACGTCGCATTTCATGATGGAGT	
CHS1-qPCR-F	CAACTCCTCCGAACTGTGT	[27]
CHS1-qPCR-R	AGCATCCAATGAAGGTGC	
LCYB-qPCR-F	CAACAAATGGGTGCTCTTCTAC	[28]
LCYB-qPCR-R	TTCAGGCTGCTGTGGTTCT	
PSY-qPCR-F	GCAACGATGCCCATACCG	[28]
PSY-qPCR-R	ACAGGCAACGACAGAGGGTG	
EXP-qPCR-F	CATCGTGAAGGTGAGCGTTA	[29]
EXP-qPCR-R	TCTTTCCGACGAATGTTTGA	
LOX-qPCR-F	GACTATTGCTACTTCTACTACA	[30]
LOX-qPCR-R	TGCATGCCTCTATCAGCTCTTC	
EG-qPCR-F	GACAACCCAAGAGCCACA	[30]
EG-qPCR-R	TGTCAGGCATCAGGTC	
βGal-qPCR-F	TGCCCAAAGCTCCCTGAAAC	[31]
βGal-qPCR-R	CCACAGCCCATCAGTGGTAA	
ACO-qPCR-F	AGATGGGCAGTGGATTGATGTG	[32]
ACO-qPCR-R	TGCATCACTGCCAGGGTTGTAG	
ACS-qPCR-F	GAAATCTATGCTGCCACTCT	[32]
ACS-qPCR-R	ATCTTGCGGATACAACTCAC	
EIN2-qPCR-F	TGGGCATCAGCAAAGGTCAA	[32]
EIN2-qPCR-R	CCACATCGCCAGCAGCAGT	
ERF1-qPCR-F	CCTACGCGGATTTCGGGTTTGA	[32]
ERF1-qPCR-R	CAGCATCCATAAATCAGCCGAG	
ERS1-qPCR-F	CGATGAAATCTATGCTGCCACTCTC	[32]
ERS1-qPCR-R	ATCTTGCGGATACAACTCACAACTG	
ETR1-qPCR-F	TTGACCGTGCCAGAAGAGAAG	[32]
ETR1-qPCR-R	AACCATCAGTCGCTGCTCAG	
SPS1-qPCR-F	GCAGGTTTATGCCGCGTATG	[33]
SPS1-qPCR-R	GCAAGTATCATGGGCTTGCG	
UCP-qPCR-F	CCCAGGCTTTACAGATAATG	[34]
UCP-qPCR-R	AGCCCTTGTAAAATGCAAGC	
POD-qPCR-F	TAAGGATGCTAAGGGAAATGATA	[35]
POD-qPCR-R	TTAATGCTGTCTCCAAAAAGTCC	
PPO-qPCR-F	TTGAGGCCAGTTTTCTTTTCTATG	[35]
PPO-qPCR-R	ATTTTACCCCTGTGTCTTTCTCAA	
PAL-qPCR-F	TGAAGCACCATCCGGGCCAA	[36]
PAL-qPCR-R	TCTCATGCAACTTTTGAGCTGCTTT	
GLU-qPCR-F	TAGGTGTATGCTATGGAAGAAATG	[30]
GLU-qPCR-R	CGAGGATGAGTTCAATGTTGG	
CHI-qPCR-F	ATGGATCTCAACGCTCCCGAAAC	[30]
CHI-qPCR-R	GTTGCTTTCCACCGCATTCAAG	

References

26. Singh, R.K.; Ali, S.A.; Nath, P.; Sane, V.A. Activation of ethylene-responsive p-hydroxyphenylpyruvate dioxygenase leads to increased tocopherol levels during ripening in mango. *J. Exp. Bot*. **2011**, *62*, 3375-3385.
27. Mei, Z.D. Study the influence by two pathogenic funguses infection on Mango *CHS* gene expression. Master, University of Hainan, Hainan, China, 2016.
28. Zhang, M.Y. Related gene mining and preliminary identification of carotene in mango fruit. Master, University of Hainan, Hainan, China, 2018.
29. Zheng, X.L.; Jing, G.X.; Liu, Y.; Jiang, T.J.; Jiang, Y.M.; Li, J.R. Expression of expansin gene, *MiExpA1*, and activity of galactosidase and polygalacturonase in mango fruit as affected by oxalic acid during storage at room temperature. *Food Chem*. **2012**, *132*, 849-854.
30. Chen, J.; Yuan, D.B.; Tan, L.; Li, F.F.; Li, Y.X.; Ai, B.L.; Zheng, X.Y.; Zheng, L.L.; Liang, D.M.; Wang, C.Z. Effects of nitric oxide treatment on the expression of softening and disease resistance related genes in mango fruit. *Guizhou Agric. Sci*. **2015**, *43*, 126-130.
31. Chidley, H.G.; Deshpande, A.B.; Oak, P.S.; Pujari, K.H.; Giri, A.P., Gupta, V.S. Effect of postharvest ethylene treatment on sugar content, glycosidase activity and its gene expression in mango fruit. *J. Sci. Food Agric*. **2017**, 97, 1624-1633.
32. Hong, K.; Gong, D.; Xu, H.; Wang, S.; Jia, Z.; Chen, J.; Zhang, L. Effects of salicylic acid and nitric oxide pretreatment on the expression of genes involved in the ethylene signalling pathway and the quality of postharvest mango fruit. *N. Z. J. Crop Hortic. Sci.* **2014**, *42*, 205-216.
33. Bai, B.B.; Geng, H.Y.; Jing, Y.L.; Zhao, Z.C.; Chen, Y.Y. Cloning and expression vector construction of *minsps1* gene in mango. *Mol. Plant Breed*. **2019**, 17, 855-861.
34. Considine, M.J.; Daley, D.O.; Whelan, J. The expression of alternative oxidase and uncoupling protein during fruit ripening in mango. *Plant Physiol*. **2001**, *126*, 1619-1629.
35. Lin, J.H.; Gong, D.Q.; Zhu, S.J.; Zhang, L.J.; Zhang, L.B. Expression of *PPO* and *POD* genes and contents of polyphenolic compounds in harvested mango fruits in relation to Benzothiadiazole-induced defense against anthracnose. *Sci. Hortic*. **2001**, *130*, 85-89.
36. Bajpai, A.; Khan, K.; Muthukumar, M.; Rajan, S.; Singh, N.K. Molecular analysis of anthocyanin biosynthesis pathway genes and their differential expression in mango peel. *Genome*. **2018**, *61*, 157-166.
